# Unseeing urban divides in Luanda and Maputo

**DOI:** 10.1177/02637758251344823

**Published:** 2025-05-28

**Authors:** Jon Schubert, Jason Sumich

**Affiliations:** 1 Division of Urban Studies, Department of Social Sciences, University of Basel, Basel, Switzerland; 2 Department of Sociology, University of Essex, Colchester, UK

**Keywords:** Urban inequality, sociospatial stratification, capitalism, protest, fiction

## Abstract

Taking China Miéville's novel The City & The City as our point of departure, we develop the idea of “unseeing” as a central cultural skill to make sense of, and live with, contemporary urban inequality. Based on long-term ethnographic fieldwork in Luanda and Maputo, we posit unseeing as a useful heuristic to capture the processes by which divisions between disparate urban lifeworlds are produced and upheld. While unseeing is a necessary, entrained social practice to live with the contradictions of contemporary capitalism, urban life also offers opportunities for moments of “breach” that reveal both the forces that reassert social division and the potential of practices that seek to force people to see rather than unsee.

## Introduction

Informal traders are an inescapable sight in downtown Maputo, as in most metropolises throughout Africa and the global South more generally. They are so ubiquitous that their presence is almost invisible and, ironically, they only become noticeable through their absence. One day, when meeting a Mozambican seasoned veteran of the aid industry for dinner, Sumich and his wife saw that the informal traders, beggars, amputees, street children, those whose presence normally fills the streets of the area, had virtually disappeared. When Sumich's wife mentioned this, their companion was bemused for a moment and then said that she had not noticed it, though now that she thought about it, it became immediately obvious. Sumich asked her what she thought happened and she just shrugged her shoulders and replied, “I don’t know, they must be periodically purged, I guess the municipality comes through once a month or something.”

In his 2009 neo-noir novel *The City & the City*, China Miéville (2011 [2009]) paints a picture of two intertwined city-states, Besźel and Ul Qoma, that exist in the same physical space but are distinct entities with separate jurisdictions and populations. Some areas of this divided space are completely in one or the other city, but many more spaces, especially in the center, are “crosshatched,” with, for example, one side of the street in Besźel and the opposite side in Ul Qoma, each of them with a different name. Citizens of these two cities thus inhabit (at least in part) the same space simultaneously, but are rigorously trained since birth to “unsee” and “unsense” citizens of the other city, preventing any accidental overlap or acknowledgment of the other city's presence. This spatial enmeshing of two divided and formerly inimical societies and polities creates a psychological and social divide, enforcing a profound separation between the inhabitants, who coexist side by side but lead separate lives in their respective cities. Besźel is depicted as drab, run-down: Ul Qoma as Byzantine in style, a “former Chairman Maoesque” state that is on a US sanctions list, but is in the midst of a blistering economic boom. Subtly registering differences in architectural style, technology, dress, or gait/habitus while immediately unseeing these “alter parts”—building, vehicle, or person—are a key cultural skill to maintain the distinct identities and sovereignties of Besźel and Ul Qoma. In addition to this, the divide is subject to draconian enforcement by the mysterious power of Breach, which cordons off areas where individuals (or non-human entities) may have breached, carrying off the offenders to places and fates unknown. To be able to navigate the urban environment successfully, their respective residents have to be both aware of the presence of the other city, while denying its existence at the same time.

The book brilliantly skewers the ways in which skyrocketing urban inequality allows the rich and the poor to live cheek by jowl, while studiously ignoring each other's existence at the same time. While Miéville, a dedicated communist and social activist, was most likely addressing urban inequality in his home city of London, we find his metaphor to be equally—or perhaps even more—apt for cities of the global south, such as Luanda and Maputo, the capitals of Angola and Mozambique, respectively, where we have conducted substantial parts of our respective, individual field research over the past two decades.

In cities like London, it is perfectly possible, in most boroughs, to walk down a leafy street of posh terraced houses, turn a corner and find yourself in a run-down council estate prowled by the “feral youth” who reside in the fevered imaginations of Britain's rightwing tabloid press and politicians.^1^ Yet, the neat spatial segregation that allows people to *actually live in separate worlds* is also made possible because routes through the city and social circles rarely intersect. In New York City, an Upper East Side life need not have any connections to a life lived forty streets further up in Harlem, let alone the Bronx or Queens. For a cosseted elite cushioned by private schools and private clubs (Gamsu, 2022), the lower classes only exist as a hypothetical concept that only irrupts in rare moments of breach—to stay in Miéville's terminology—even though most of the essential services that make this pampered lifestyle possible are run by scores of low-waged, often illegalized, migrant labor. One could also argue that the parable of *The City & The City* applies to Jerusalem, perhaps the most obvious real-life example of two (claimed but contested) capital cities and sovereignties co-existing in the same geographical space—though as effective Palestinian statehood seems, for now, reduced to what Eyal Weizman (2007) aptly terms “checkpoint sovereignty,” socio-political segregation is there, spatialized via separation walls, the encroachment of illegal settlements, and at the barrel of a gun.^2^

Riffing off Miéville's thought-provoking image of two cities that occupy the same physical space but exist countries apart, we seek to think here not so much about competing sovereignties, but to extend this idea to gain a fresh handle on the socio-spatial processes that enable “unseeing” the inequalities of contemporary urban socio-economic segregation.

Urban segregation and “economic apartheid” (see ethnography below) based on some form of “unseeing” can be found the world over. Examples abound, but briefly returning to London, this entails the construction of political and social infrastructure that allows the super-rich “hide in plain sight” (Knowles, 2022). While they are thus not unique in terms of socio-economic stratification, we argue that Luanda and Maputo offer a useful prism through which we can conceptualize such processes of unseeing. If the roots of the British class system can be said to trace back to 1066 if not earlier and equality was never the founding virtue of a monarchy, constitutional or otherwise, both Luanda and Maputo experienced what we could term “compressed history,” and, at least in principle, an aspiration to egalitarianism.

As with many cities the diversity of the inhabitants of Luanda and Maputo is staggering; the descendants of slave traders rub shoulders with those whose ancestors were stolen, those who felt the full force and/or were complicit with colonial domination share streets with those whose grandparents were only vaguely aware they lived in a colony until the final decade before independence. However, in the relatively short time span of the past 60 years, residents of Luanda and Maputo were at the focal point of colonial efforts of authoritarian, racial capitalist developmentalism, struggles by victorious anti-colonial liberation movements to build a classless Marxist-Leninist society, economic collapse brought on by civil war, and the flourishing of what is called in both countries “savage” capitalism under dominant ruling parties whose democratic veneer has increasingly failed to mask deeply authoritarian practice. Not only have all these events occurred in such a short time span, but all of these transformations can be traced back to many of the same people and families (Schubert, 2017; Sumich, 2018). The ability to unsee the poor in contemporary Luanda and Maputo rests comfortably aside a popular socialist nostalgia, many wax poetically about the noble, but ultimately doomed cause to build an egalitarian society for those who they no longer see at all. It is this seeming disjunction, still fresh in living memory, that animates the dynamics of unseeing in Luanda and Maputo.

We focus here on how “unseeing” is a central facet for making urban life bearable, comprehensible. We seek to demonstrate this central feature of contemporary urban life by way of two African cities, Luanda and Maputo, that were once seen as showcases of “Africa rising” and are now sinking back into the morass of economic crisis. Angola and Mozambique share a Portuguese colonial past, a painful liberation struggle, and a legacy of state-socialism, single-party-state, and brutal civil war. Now both nominally democratic, both countries are still governed by their respective liberation movement-turned-dominant party, the Popular Movement for the Liberation of Angola (*Movimento Popular de Libertação de Angola*, MPLA) and the Mozambican Liberation Front (*Frente de Libertação de Moçambique,* Frelimo), both facing eroding popularity and legitimacy but abusing the full privileges of incumbency to stay in power.

Based on our experience of living in and working on the capital cities of Angola and Mozambique, respectively, we argue that dynamics that recur across the globe—in local specificity and contingency—are prefigured more starkly and clearly visible in places like Luanda and Mozambique, where the separation between lifeworlds less neatly spatialized and contained than in, say, London's council estates, or Paris's *banlieues*. In cities where the interstitial spaces between new gated communities are “invaded” (in the terms of city planners) by “informal” constructions, or where drivers and passengers of large SUVs can choose to either purchase anything from soft drinks to dog leads from informal roadside vendors, or zip by in blissful, climate-controlled ignorance, blindness is an engrained, continuous, if precarious, social practice. The two cities thus represent differentiated components within the long and uneven history of global capitalism; evidently, the unseeing of urban divides will be differently configured across the space-time continuum of modern capitalism.

In this instance, we develop our argument first by looking at what “unseeing” enables us to understand in contemporary urban life that existing approaches to questions of urban inequality and stratification may miss. Rather than a full-fledged theoretical apparatus, we posit unseeing as a useful heuristic to capture the processes by which divisions between disparate but spatially enmeshed urban lifeworlds are produced and upheld. Following this brief discussion of our conceptual positioning, and how we develop Miéville's ideas in conversation with other notions of urban socio-spatial stratification, we unfold our article mirroring the structure of Miéville's book: Besźel, to explore unseeing practices by the city dwellers connotated with the lower classes; Ul Qoma, for the upper classes; and finally, Breach, that limited in-between space where separation is breached.

We base our essay on our individual ethnographic material collected in both cities, roughly over the past two decades and our shared love of Miéville's book. As a side note on method and positionality: ethnographers would like to think of themselves in the field like the foreign researchers that appear in *The City & The City* and become central to the book's plot—someone who has studied the ways of being so closely that he is able to walk “with equipoise” between the two worlds, belonging fully to neither:

*How could he do it? Walk like that?’ ‘He's been a student of the cities,’ Ashil said. ‘Maybe it took an outsider to really see how citizens mark themselves, so as to walk between it.*

*[Miéville, The City & The City, 368]*

In practice, however, our work of suspended immersion probably resembles more the figure of the tourists who get an entry visa to Ul Qoma or Besźel after a two-week crash course in unseeing. The training is incomplete, and their bodily entrainment of unseeing far from naturalized, so from the perspective of inhabitants (and Breach) they will get a lot of things wrong, which will only not be sanctioned by Beach because of their status as a bumbling foreigner. But failing to unsee properly does allow us some glimpses across the divide that usually keeps the two realities separate, allowing us a modicum of insight that might perhaps be closed off to someone fully habituated into the system.

## Unseeing urban divides

*If someone needed to go to a house physically next door to their own but in the neighbouring city, it was in a different road in an unfriendly power. That is what foreigners rarely understand. [Miéville, The City & The City, 86]*

While much has been written about “right to the city,” outside of expressing moral condemnation of the ever more polarizing inequalities that characterize contemporary urban centers, far less attention has been devoted to the “unseeing” as a condition not only of class consolidation, but of social survival. It is exactly the social underpinning of “unseeing,” the ways in which it is intertwined with issues of stratification, exclusion, and the evolving meaning of urban citizenship that we intend to explore.

In the past few decades, many urban scholars have focused on the increasingly enclaved nature of cities, what Mike Davis described as “a new form of class war whose medium is the built environment (Davis, 1990: 228). The combination of changing forms of security, the rise of private “armed response” and a regulatory environment that explicitly favors the privileged have allowed the lucky few to shield themselves from the consequences of growing social polarization (Caldeira, 2000; McKenzie, 1994). In the African context, the enclaved model is often conceived as a sort of “back to the future,” where neo-liberalism and its associated brutalities have supercharged older forms of exclusion with roots in the colonial period (Murray, 2011; Nielsen et al., 2021). These emerging forms of segregation can take the form of an ad-hoc patchwork of securitization, carving out highly individualized spaces for the privileged as Paasche and Sidaway described for central Maputo (2010: 1571). Or it can involve complicated, subterranean negotiations with foreign capital to construct gleaming new cities for the elite (Côté-Roy and Moser, 2019; Murray, 2015; Watson, 2014). These forms of securitized segregation are profoundly reshaping cities across sub-Saharan Africa and the wider world.

Such physical separation is, however, often performing detachment of lifeworlds that are, in fact, “entangled” (Appel, 2012; Sumich, 2023). Walls produce an impression of separation which serves the purpose of cloaking the fact that enclaves are utterly entwined with the wider world; they are two sides of the same coin. Building on this insight, we seek to explore the ways in which a situation seemingly characterized by separation is profoundly entangled, or “crosshatched,” in Miéville's parlance. Spatial separation is an illusion, based on a form of “capitalist realism” that is ultimately completely unrealistic. Keeping lifeworlds apart depends not on physical segregation, but on conceptual one, the ability to “unsee.”

In cities like London and Paris, there is both spatial segregation (council estates and *banlieues*) and the social, political, and media infrastructure (Knowles, 2022) that both invisibilizes and normalizes the presence and influence of the super-rich. In Luanda and Maputo, despite attempts at enclaving, lifeworlds are physically more enmeshed and the contrasts more readily visible. Despite real and visible practices of socio-spatial segregation in the metropoles of the global North, unseeing is therefore much more engrained in cities of the global South, where grotesque wealth and abject poverty coexist in the same space and interact in quotidian ways that call for the simultaneous negation of social coexistence. And this crosshatching—which is, across different urban contexts, driven in no small part by foreign capital and elite collusion—is more evident in cases like Luanda and Maputo than it is in London.

To successfully navigate the city, one must be able to register the presence of the hundreds of thousands of people who make up the so-called “informal economy,” whose presence runs the gamut of providers of vital services to a serial annoyance. At the same time, to conceptually operate in a situation of such stark inequality, one has to constantly, routinely and automatically, unsee them. To inhabit the city as if the vast majority of its population exist in an entirely different reality with which you shall never interact requires a special kind of entrained, pre-cognitive, almost bodily habitus that, we would argue, echoes Miéville's inculturated practice of unseeing.

In recent years, many scholars have explored the concept of ignorance not as a lack of something, but rather as socially generative and a key factor for the exercise and expansion of power (Bovensiepen and Pelkmans, 2020; McGoey, 2007; Proctor, 2008). David Graeber (2012) has argued that power is not expressed through knowledge but is manifested through ignorance. Power is the ability not to have to know anything about those below you, while the subaltern must study the whims, habits, and foibles of the privileged intently to survive in a context characterized by stark inequalities. A parallel conversation in critical security studies or from work on toxic exposure speaks of “strategic unknowing,” analyzing how ignorance is socially produced and deployed strategically to eschew political responsibility (Gould and Stel, 2022; Shapiro, 2014; Ureta and Otaegui, 2021). This is similar to what McGoey calls “a ‘will to ignorance’ within regulatory bureaucracies which works to circumvent a regulator's ability to carry out its explicit aims and goals” (2007).

There is much debate, though, concerning the role of conscious deployment in regards to ignorance. Bovensiepen and Pelkmans have argued that a way forward is the concept of “wilful blindness” which “can imply both strategic (non-) perception and normalized disposition” (2022. p. 388). Bovensiepen and Pelkmans insightfully point to the ways in which political ignorance is more of a process than an outcome, even if there is the danger of some conceptual slippage as the word willful entails conscious or deliberate decision making by definition.

However, while concepts such as “strategic ignorance” or “wilful blindness” require extensive mental gymnastics for the careful manipulation of information and understandings of the social order, “unseeing” is the result of a deep internalization of the social order the enforcement of which is largely left to the individuals themselves. Strategic ignorance is above all, strategic, and willful blindness focuses on deliberately ignoring uncomfortable information in favor of “the stories those in power tell themselves to legitimise their actions towards themselves” (Bovensiepen and Pelkmans, 2020: 396). Unseeing, we suggest, is much less of a strategic choice, but rather an almost automatized consequence of class habitus, through which the existence of the “other half” is bracketed out. In that sense, the difference between willful blindness and unseeing is akin to what differentiates emotion and affect. Emotion requires articulation within a social framework; affect is a precognitive, almost somatic reaction (Thrift, 2004).

That, we suggest, is the main difference from the colonial period to the present in Luanda and Maputo: during colonialism, society was very openly bifurcated between the worlds of the colonizer and the colonized, with both being hyper-aware of each other's presence, the “nervous conditions” of colonialism premised on coiled resentment and the constant undertow of violence (Dangarembga, 2004; Fanon, 2002; Hunt, 2016). While the Portuguese colonial regime deployed a rhetoric of multiracial harmony and dubbed its colonies “overseas provinces” to resist any calls for independence, no one could credibly claim unity between the white colonizers and the largely Black colonized population. After independence, both Angola and Mozambique embraced socialism as a way to create the “New Man,” and a classless society freed from the shackles of colonial oppression, encompassed in, for example, the MPLA's slogan,—still deployed today—*o povo é o MPLA e o MPLA é o povo* (the people is the MPLA and the MPLA is the people). Sentiments in Mozambique were very similar. However, both the civil war and the reality of being embedded in circuits of global capitalism soon put paid to this ambition. With the advent, and then feverish embrace, of rapacious capitalism society was deeply bifurcated again, this time less along lines of race but rather of class. *Unseeing*, as an entrained way of navigating urban life, becomes a key way in which to live with this bifurcation when inequality is glaring but notions of political legacy and nationhood still rest on the presupposed unity of the elite and “the people.”

We advance three theses on unseeing:

1. Unseeing is an apt heuristic for the entrained, engrained practices of invisibility that define contemporary urban life under capitalism, as it is an essential social practice for urban residents, in different ways both for the haves and the have-nots, to make urban inequality bearable and livable.
2. This practice has become so engrained, that it makes it difficult to actually *see* the world around us. Not just in Maputo and Luanda, one may diffusely sense a lifeworld different from our own but almost immediately unsee it: upholding contemporary capitalism requires a lot of unseeing (e.g. climate change, labor exploitation, deadly Frontex border pushbacks etc.).
3. There are moments of accidental and deliberate breach that reveal both the social forces that reassert the division and the potential of practices that seek to force people to *see* and register rather than constantly unsee.

## Besźel

During Schubert's fieldwork in Luanda in 2011, the second (and last, to date) Luanda Art Triennial took place, organized with prominent backing by Sindika Dokolo, Isabel dos Santos’ husband (and thus then President dos Santos’ son-in-law). Sitting down over lunch in the *quintal* (courtyard) of his rented accommodation with his friend Alberto, a young community activist from the nearby Cazenga high-density, informal neighborhood, Schubert asked Alberto whether he had attended any of the events or public exhibitions of the Triennial, as they were free and open to the general public. Smiling dejectedly, Alberto said:

It's open to everyone, yes. But not everyone will go, because in their subconscious they will know that it is not for them, that it's only for the elites. Recently, I was at Chá de Caxinde [a downtown cultural venue], and by chance was there for the launch of [famed author] Pepetela's most recent book; I was there for something else, but I just happened to be there. I felt immediately that it was not for me; not only because of the prices at the bar. It's also a question of skin color. Someone like me, who catches dust all day and is more rustic will feel out of place. Also, they have strange ways of conversation. It is a social and economic Apartheid. We want to tear this wall down. But the problem is that everyone just wants to get up, and once they are on top, they forget about the ones down below.

Their conversation moved on to a recent speech by (then) President José Eduardo dos Santos, who had been ruling Angola since 1979, in which he had rejected any responsibility for widespread levels of poverty in Angola, by saying that poverty already existed during colonialism, when he was a child—a speech that was much ridiculed by Luandans. Alberto, however, thought dos Santos’ speech was in many ways more honest than his usual, scripted speeches: “This speech revealed two truths: First, this man does not know the reality of the population. Second, he does not respect the *povo*.”

This was the period of “peak dos Santos,” when the official exchange rate for the Angolan kwanza was artificially pegged to the USD, and Luanda regularly topped rankings as the world's most expensive city. Especially in the period leading up to the 2012 general elections, pronouncements by dos Santos or other government officials from the ruling MPLA party increasingly sounded like affirmations of an alternate reality. This betrayed either the regime's boundless cynicism or, to many, their actual ignorance (or both) of the lived reality of a majority of Angolans. The MPLA's historic revolutionary slogan, *o MPLA é o povo e o povo é o MPLA* was trotted out again for the campaign but had rarely sounded so hollow.

But while elite statements appeared as the clearest evidence of parallel, yet profoundly disconnected urban realities, we would argue it takes a particular kind of blindness to uphold the “economic apartheid” that Alberto was referring to. Young, middle-class urbanites paying their household employees a smidgen above minimum wage and giving them not just one but one-and-a-half days off per week, or offering them pens and used clothes for their children could cast themselves as the good guys in the script of their upwardly mobile life stories while perpetuating the exploitation of cheap labor that sustained their lifestyle.

This is, evidently, at city level, a fractal image of global processes of labor and tax arbitration that perpetuate the economic dominance of the global north at the cost of ecological and economic exploitation of the global South.

*…still the traditional baroque curlicues of Ul Qoma's heritage sights were made almost pitiful by their giant young neighbours. Like all Besźel dwellers, I had become used to shopping in the foreign shadows of foreign success. [Miéville, The City & The City, 162]*

As in this quote, Alberto and other less privileged urban residents in cities like Luanda or Maputo have had to learn to “do their shopping” or more generally lead their lives in the shadow of a boom predicated on the rapid influx of foreign capital. While huge flows of foreign, unaccountable money have also transformed citiscapes and property markets in cities like Geneva or London, zoning laws and building conservancy regulations make their impact more easily unseeable—an apt illustration would be the underground extensions of listed houses in Hampstead or other affluent London boroughs. In Luanda and Maputo, largely unfettered by laws and regulations, elite-connected speculation and property development have over the past decade radically reshaped city skylines. New, glass-paneled towers dwarf the colonial-era modernist, now by comparison modest-looking, apartment blocks, displaying the kind of aspirational, generic Dubai modernism typical of “world class city” ambitions. And while it is tempting to dismiss these developments as purely speculative fantasies (de Boeck, 2011; Watson, 2014), Ricardo Cardoso convincingly argues that talk of “fantasies” obscures politics of planning and designing and the circuits of finance and expertise that bring about specific forms of urban development (2016).

In our cases, boomtown urbanism is a tool of symbolic power and manifestation of structural violence. New urban developments encapsulate the promise of economic growth and modernity, and postwar reconstruction, and the hegemonic ambitions of the regime to impose its vision of the country's future onto the cityscape (Schubert, 2015). Ordinary residents did and still do look to these aspirational projects made concrete in a mixture of longing and awe. For a long time, in Angola, the promise of material betterment by aligning with the political status quo was one of the “weapons of the regime” (Schubert, 2018), which worked even as it was very clear that the amenities and services offered by these new developments were only for a certain, very specific kind of citizen. In that sense, navigating the changing urban landscape requires simultaneous sensing of prosperity and possibilities of advancement and unseeing of the kinds of ostentatious, rapacious, conspicuous consumption that marked the boom years in Angola and Mozambique.

But there are instances where elite lifeworlds irrupt in the physical environment, connotated with the “informal,” unplanned city of poorer residents:

*When an Ul Qoman stumbles into a Besź, each in their own city; if an Ul Qoman's dog runs up and sniffs a Besź passersby; a window broken in Ul Qoma that leaves glass in the path of Besź pedestrians — in all cases the Besź (or Ul Qomans, in the converse circumstances) avoid the foreign difficulty as best they can without acknowledging it. Touch it if they must, though not is better. Such polite stoic unsensing is the form for dealing with these protubs — that is the Besź for those protuberances from the other city. [Miéville, The City & The City, 79-80]*

Maputo and Luanda were designed by colonial planners and administrators as bastions of separation, where race, class, and occupation were spatially fixed (Gastrow, 2020; Morton, 2019; Schubert, 2020; Sumich 2012, 2018). While the end of colonial rule deracialized the spatial hierarchies of Maputo and Luanda, it did not necessarily democratize them. At independence old established urbanites and an emergent middle class fled the Besźel of the *bairros* for the Al Qoma of the central, concrete city, the former citadel of the colonists (Tomás, 2022). With the privatization of the housing stock this spatial hierarchy began to break down (Sumich and Nielsen, 2020). Skyrocketing real estate prices, eye-watering rents, and limited space in the city center has meant that the privileged, especially those too young to directly benefit from the great post-independence privatization of housing, have been forced to spread into the *bairros* and the outskirts of the city, another symptom of what is an increasingly enclaved social and economic model. It is now common to see high walls guarding the swimming pools, carefully manicured lawns, and spacious houses of the upper middle class rising alongside the zinc-roofed houses of the poor.

Both of us have often visited research interlocutors of the emergent or the more established middle class who were in the midst of building houses in what was once the outskirts of the city or in new extension zones. Such visits invariably involve a drive of 40 min to an hour (and this is with minimal traffic). In Maputo one has to pass through toll booth after toll booth, and in Luanda, Brazilian-built turnaround after Brazilian-built turnaround on the main traffic arteries, and it can take hours to get to work on a busy weekday. The landscape is primarily made up of small, self-built houses made out of rough concrete, but occasionally a protuberance of gentrification appears, a fancy restaurant or an upscale bar.

The houses that are the most obvious protuberances of wealth in such an extension zone of the city are massive, or at least they will be when they are finished. The huge amount of labor needed to complete these houses does not end with the simple physical construction. Many are in areas with little or no municipal services. Stories abound about endless rounds of negotiation with municipal officials, the need to make use of every facet of social and cultural capital available to try and persuade officials to create a connection to the power grid, water, and sewage.

While the privileged sections of Luanda and Maputo's population have been spatially dispersed, outside those who were able to sell at a profit and had somewhere else to go, this development has brought few benefits for the wider population. Infrastructure and services have instead become increasingly individualized, with one house completely connected to the network while the neighbors must all make do as best they can. In many ways, these well-serviced, walled protuberances might as well be invisible, as there is little to no social, infrastructural, or material connection to the surrounding area. Here, despite spatially rubbing shoulders, distinct modes of housing and living coexist in the same physical space, requiring constant unseeing across the social divide.

## Ul Qoma

Returning to the opening vignette of this article, we now turn to the practices of unseeing and unsensing practiced by the upper classes. For example, one Mozambican professional told Sumich that she was plunged into a deep depression when she had to visit a *bairro* (poor neighborhood) for work. Even though it was only a short distance from her house, she had never been forced to notice the existence of her neighbors before, nor the conditions that they lived in. Negotiating daily life in Maputo without “unseeing” proved to be deeply traumatic. Despite the daily influx of maids, security guards, waiters, gardeners, informal traders and hundreds of thousands of others who stream into the social islands that form the archipelago of privilege every day, in many ways the city center and the privileged enclaves of Maputo a float like a balloon, ever so lightly tethered to the body of the city below.

Similarly, living a life of privilege in Luanda requires a lot of constant unseeing, a lot of which is to do with patterns of consumption. Regularly during fieldwork, Schubert felt he was passing from one city to another in a matter of mere minutes. Even when the different classes live seemingly cheek by jowl in the city, the divide seems unbridgeable. The inner-city neighborhood of Bairro Operário (B.O.), the famed “cradle of Angolan nationalism” (Moorman, 2008) is separated only by one main thoroughfare from the airy, elite residential area of Miramar, home to many embassy residences; the furthest end of Miramar, where the former First Lady's palace stands, overlooks the informal neighborhood of Boa Vista (“good view”), where self-built cement block houses teeter precariously on the steep sandy slope leading down to the Port. Spending a morning at a Church service in a boggy side lane of the B.O., where the roughly 100 congregants managed to collect the equivalent of USD 100 during offers, Schubert would cross Rua Nduduma to spend an afternoon by the poolside with imported champagne and nibbles that would cost a multiple of what this morning's congregation had sacrificed their meager incomes for. Or consider an afternoon of leisure amongst what could be termed an incipient middle class—salaried professionals who came of age after independence and often hail from party-connected families, such as university lecturers, meeting up at a private house in a new, planned expansion area in Luanda Sul. Here, too, alcohol and grilled meat are abundant, and more precarious urban lifeworlds seem very far away indeed.

In such areas of privilege, the only irruption of the “slum” are warnings of “marginals” sneaking up to assault honest citizens and burglarize their houses (though actual instances of violent crime in Miramar were for most of the time, relatively low). In the everyday routine, these two lifeworlds coexist without much interaction—despite instances of spatial emmeshing as we show in the following.

Several times during fieldwork, Schubert was taken to Mussulo, the peninsula off Luanda, where those who can afford it flee from the heat and the hustle of the city on the weekends. In the 1980s, there were only a few *palhotas* or fishermens’ cabins on the island, but now it is full of massive villas with their own electricity and freshwater supply. Spending a weekend at one such “beach house,” friends of the family stopped by for lunch, arriving in their white motor yacht, and bringing a fish dish, and an ice cold Magnum bottle of Cristal Roederer champagne. With such a bottle costing easily double the nominal minimum wage (USD 100 in 2011), the party casually drank it as *apéritif*, with honeyed peanuts and pistachios. The lunch was copious, and besides the fish, there was roast beef *and* grilled chicken, some salads, and Argentinian Malbec.

Here, the separation between two lifeworlds seems neatly spatialized, but, as mentioned earlier, this life of leisure depends on the unseen presence of household employees, the many *empregados,* who make things turn, prepare the food, fuel, and crew the boats and clean the houses. And while the relaxed setting might allow for some jocular expressions of seeming familiarity across the divide, it only goes so far—and remains strictly one-way, with the boundaries between *patrão* and servant strictly upheld in this game of seeing while unseeing.

Even when the separation is not as clearly spatialized as on a relatively secluded island, it would appear that some things have not changed fundamentally since the end of colonialism. This is, in Angola and Mozambique nowadays, not so much primarily a racial divide, but a class divide (though that divide is often expressed through racialized idioms, see Schubert, 2017; Sumich, 2013), between those who have, and those who have not; those who are educated and those who are not; and those who command and those who obey: *quem manda, manda, quem não manda, cumpre* (those who command command, those who don’t command obey). While some members of the lower classes are roped into the service of upholding a privileged lifestyle, that life works very well without acknowledging the existence of the majority of the people who dwell in the “informal” economy.

## Breach

What are the social mechanisms that enforce this habituated separation from upper-class lifeworlds that Alberto alluded to? As with Miéville's two cities, these mechanisms must perform a dual role of intimate connection and “unseeing” simultaneously. This double function was illustrated when Sumich once took Jonas, the *empregado* (domestic worker), who worked at the house he was staying at, out for drinks. Jonas had perhaps one too many and insisted on taking Sumich to the neighbor of his employer, a family that was well-connected to the upper reaches of the ruling party. He pounded on the door and when Tomás, the owner of the home, opened it, he vehemently told him “see, I told you I have a friend this color,” all the while pointing at Sumich. Tomás looked somewhat bemused, he told Jonas to hush and then sent him to another room to tend to his mother, and invited Sumich into the living room for whiskeys. After 20 minutes or so, Tomás then told Jonas to go out and fetch some cokes (despite the fact that Jonas did not actually work for this family) and then he could leave, while Tomás and Sumich continued their chat. Jonas had worked next door to Tomás for 13 years, he knew the family, he had tended to their parents and their children. In many ways, their lives were intimately connected and as such Jonas was definitely “seen.” However, this form of intimacy was also predicated on being invisible, not as a presence *per se*, but as a person who must exist, unseen, in the background. When, after one drink too many Jonas tried to enact a form of breach, he was quickly banished from the picture, again relegated to the realm of the invisibilized other.

On the one hand, *empregados* could be seen as the living embodiment of “breach” as their presence is ubiquitous: every family of almost any means employs at least one household employee, and this employment is often built on long-standing family relationships and numerous forms of moral and social obligation—obligations that, northern European expatriate *patrões* sometimes seek to deny under the pretense of egalitarianism, as Flora Botelho (2021) elegantly demonstrates. On the other hand, despite the dense social relations that undergird this form of employment, they are the epitome of “unseeing” confined to a social role that means they can often be largely invisible. Thus, Jonas's attempt to “breach” was not a success. While he may have momentarily been “seen” by his neighbors, it was not so much in his capacity as a person but rather as a function, that is, as *empregado*, and he was almost immediately sent off to the kitchen to fulfill his accustomed role. “Unseeing” is not restricted to rendering sections of the population invisible, although this does occur, but also through the medium of social class. Thus, while *empregados* are ever present, the subservience baked into their social role allows them to fade into the background of daily urban life. This also points to an extension of Miéville's initial idea: separation of lifeworlds exists not only from one street to the next, but also within the home itself.

This form of “unseeing” by the upper classes, however, becomes far more complicated in relation to members of a group who have both generally low social status, but are not required to necessarily be subservient—such as the police. Like *empregados* the presence of the police in the city center and upper middle-class neighborhoods is also ubiquitous, but for many of those we know, all too visible. In Maputo, the more privileged urbanites tended to view them with contempt, one of the few things they have in common with the wider urban population. Once when Sumich was riding through town with a Mozambican professional who was pointing out that the police all had a new style of uniform, which was supposed to symbolize a transformation in the “corporate culture” of the institution. The professional found this laughable, as he said, “it would make more sense to change job conditions rather than the uniform and look, they do not even have enough money for all the police to get the new uniform. Just the boss has one, that is pretty much symbolic of the wider problems.” Such contempt also mingled with pity: a stand-up comedian once described the police as “country bumpkins” who are so thin that the weight of the rifle slung over their shoulder causes them to bend towards the ground and implored the audience to treat the police's notorious demand for bribes as the pathetic expression of poverty.^3^

Underlying both pity and contempt, though, was unease, and everyone in Luanda and Maputo has had unpleasant experiences with the police. In addition to routinized violence against especially female street vendors, the most frequent occurrence was probably trying to avoid the constant demand for bribes that follows routine traffic infractions, real or imagined, and the delicate negotiations that police roadblocks often entail. In many cases, the privileged can rely on social capital to help resolve any unpleasantness. Sumich once witnessed this when offered a lift home after a dinner party in Maputo. He had forgotten his ID and when stopped at a checkpoint, the police were soon intimating that without some form of payment, he would be arrested. The driver, who the police soon found out was related to a high-ranking official, demanded that the police accompany him and he walked a short distance away. After what seemed from a distance to be a very charged conversation, the police returned and muttered something and they were able to continue our journey with no further difficulties. Both authors have heard many similar stories about using a family name or (pretend) connection to get out of trouble (Schubert, 2019).

While this does entrench the overall pattern of social hierarchy mediated through the invocation of kinship ties, it is a constant negotiation and many initial demands for deference have the overall effect of dramatically escalating the situation. Both authors know of more than one person who had to be rescued from a police station when a demonstration of social capital went horribly awry. Nor is this based entirely on demands for bribes. In Mozambique, it is widely assumed that the police and security services are complicit in the two major waves of kidnapping for ransom, largely targeting minorities such as those of Indian descent and more recently Turkish businessmen that have erupted in Maputo. Most members of Maputo's middle class view the police with pity and contempt, but this is combined with a wider sense of menace. As one professional remarked to Sumich, “they may be lazy, but their guns are heavy with bullets and they shoot them to lighten the load.” The best way to deal with the police in Maputo and Luanda is thus to try and ensure the police “unsee” them.

There are numerous strategies for this, such as driving cars normally associated with high-ranking members of the ruling party in the hope that the police think you were part of the *crème de la crème*. While Schubert did not have his own car during fieldwork in Luanda, when driving a borrowed car or riding along with friends, there was always a notable difference between a car that constituted a legitimate target for harassment by the police and a car that signaled privilege and power.^4^

But in interactions with the police, for a brief moment the normal social hierarchy can be turned upside down and now it is the privileged (if not the top rungs of the social ladder who are largely insulated) who endeavor to escape the attention of those they would never normally have to acknowledge. The best way for most people to deal with the police is to pretend they are not there, to “unsee” them, while also striving not to attract their attention in any way. This precariousness is likely, in part, a factor in the middle-class preference for private security, where the unpredictable power of the police can be tamed, and security ensured through what is essentially a deferential *empregado* with a gun.

If the police and *empregados* form the backbone of the mundane, day-to-day forms of breach, riots, and incidents of urban rebellion are emblematic of the spectacular, when the social order visibly stumbles. James Holston describes how in São Paulo taggers from the periphery mark seemingly inaccessible surfaces with their repetitive graffiti script, “to create a new visual public of city surfaces that people *cannot avoid seeing*” (Holston, 2009, italics ours), reading this as expression of a new, defiant form of citizenship. Similarly, Jaime Amparo Alves, writing on police violence in São Paulo's *favelas*, describes how the mothers of black youths killed by the police reappropriated the Patriarch's Square in the center of the city, investing this space of normative, white urban civility with their bodies to denounce the nation as an anti-black project and politicize black deaths in the usually invisibilized margins of the city (2014). And when, as in Rosalind Frederick's ethnography of waste management in Dakar, crumbling public infrastructures are devolved onto laboring bodies, their refusal to work and the ensuing pile-up of refuse in the city embodies “workers’ refusal to be the castaways of society” (2018: 131) Extending this to Miéville's notion of Breach, we suggest that a main function of urban protests is precisely to force the other half to *see.*

In Luanda and Maputo, too, urban protests and “riots” have become a semi-regular occurrence, despite a hardening of authoritarian tendencies in both countries, with major uprisings breaking out in Maputo in 2008, 2010, 2013, and 2024/25 (Bertelsen et al., 2014; Sumich, 2018) and regular street protests in Luanda since 2011 (Blanes and Samussuku, 2022; Faria, 2013).

In 2022 Maputo, as with so many other places in the world, was rocked by the “cost of living crisis” and again as with so many other places, the response of the government and ruling party demonstrated that they viewed their population with something between contempt and utter indifference. In July, bus drivers and *chapistas* (the drivers of mini vans known as *chapas* that serve as the primary mode of public transport in Maputo) went on strike, effectively shutting the city down. Many middle-class urbanites soon started sharing with Sumich anonymous WhatsApp messages that called for a general strike. They further demanded that people burn down popular eating establishments, key service providers, and local markets for not obeying the strike and ended with the threat of killing one police officer per week until the government helped the people. During the strike, there were a few incidents where residents of *bairros* near freeways stoned the cars that passed by. However, outside of jokes wondering how many thousands of police would have to be assassinated before the government was forced to notice, much less care, violence was extremely limited. Apart from staying at home to avoid the freeways and grumbling about the fact that the public transport strike meant their *empregados* could not get to work, the privileged were largely unaffected. While the bloodcurdling threats being posted on social media were fun to share and speculate on, it proved to be quite easy to “unsee” an anonymous threat and go on as before. Despite the drama of these uprisings, they seem to have been quickly re-absorbed as part of the urban social fabric, something that is yet another inconvenience, while also adding some spice and excitement to what otherwise would be a humdrum day. The respite may have been short-lived, as Frelimo claimed victory in a presidential election widely seen as fraudulent, both in Mozambique and internationally, and the resulting protests have left over 300 dead, and schools and shops closed, with no real end in sight. Despite growing popular contestation though, it is not clear how the current social order would be fundamentally transformed no matter who assumes the presidency.

As in Maputo, social media allows for a bit of temporary breach, or rebellion through proxy, in Luanda, too: while under former president dos Santos, a leaden silence had plumbed more rebellious forms of expression, rising comedy stars now poke fun at politics and the history of the civil war. And in protest against the dire living conditions, the poor quality of public services, and the “death of their dreams,” young Angolans in 2018, living in the poorer, informal neighborhoods of the city, started a campaign on social media, which quickly went viral. Under the laconic hashtag #acabademematar, that is, “*acaba de me matar*” (just kill me already), they posed for photos laying on the ground, their heads and torsos seemingly crushed or submerged by items as varied as butane gas bottles, furniture and consumer electronics, cinder blocks, schoolbooks, or the stagnant water pools and rubbish heaps that mar most of the informal *bairros* of Angola's cities. This was an attempt to force the well-to-do, and by extension city authorities, to *see* the dire conditions under which a majority of urbanites had to live. In the way the hashtag was circulated and gained traction, we could see incipient instances of solidarity across the Breach. Yet any more forceful challenge to the status quo is quickly absorbed back into the usual separation. In Luanda, it proves in most cases still relatively easy to depict protest attempts as “disorderly confusion” caused by the “uneducated masses” that need not affect the lives of the middle classes still living in relative comfort in the current *status quo*.

## Conclusion

*It's not just us keeping them apart. It's everyone in Besźel and everyone in Ul Qoma. Every minute, every day. We’re only the last ditch: it's everyone in the cities who does most of the work. It works because you don’t blink. That's why unseeing and unsensing are so vital. No one can admit it doesn’t work. So if you don’t admit it, it does. But if you breach, even if it's not your fault, for more than the shortest time … you can’t come back from that. [Miéville, The City & The City, 370]*

We have focused on a particular kind of unseeing, one that allows those who gained their privileged positions through a Marxist revolution that was dedicated to giving birth to a new, egalitarian society, and the following generations who would supposedly transcend the divisions and cruelties of the past, to largely ignore the yawning gap between them and their fellow citizens. This is simply one instance of a form of social production that has increasingly, if in very different ways, become the basis of contemporary urban life. From the climate crisis to the deliberate killing systems erected at the outside borders of the EU and the US (de Leon, 2015; Tchilouta et al., 2023, e.g.), to an economic system based on the illusion that finite resources can provide infinite growth and that values revenue over lives, capitalism trains us to unsee the horrors upon which privilege is predicated.

This realization remains vitally relevant, especially when large sections of the population simply “unsee” all the evidence contrary to the day-to-day assumptions that allow them to go through life without being trapped in an unending moral crisis, much less to acknowledge the urgency and utter absurdity of the crisis. This is why activism, to be meaningful, has to breach, to force people trained to unsee to finally *see*—with Extinction Rebellion glueing themselves to the street or throwing soup at canonical paintings as just one obvious example.

Much as late capitalism, in many ways, *The City & The City* is a story of a gigantic fraud. While the lives of the privileged may be all but invisible to the poor, a completely different and inaccessible world that exists 300 meters away, that does not mean the poor are not painfully aware of the existence of the privileged, even while knowing the gates that guard privilege are permanently shut to them. The privileged on the other hand, may be more or less unaware of the existence of 80% of the wider urban population, but their lives are still structured by all-encompassing, if in some ways, nebulous threats ranging from life and limb to peace of mind. The very act of unseeing also ensures that the unseen is always there.

Unseeing then is a useful heuristic to think about processes of social segregation in contemporary urban life, the entrained practices that navigating highly unequal, economically but not spatially segregated cities required of its residents. As we see here, the segregation of lifeworlds permeates all urban life, including the intimacy of the home, making the entrained habit of unseeing starkly visible as a vital everyday practice to navigate urban divides—although, as we have shown, it is often more unidirectional than in Miéville's case: those on bottom see quite a bit, while it is the privilege of power to be entrained to habitually unsee. Unseeing, in that sense, might be a useful analytic with which to understand the interplay of seeing and unseeing in fundamentally segregated cities elsewhere. While we developed our argument via Luanda and Maputo, unseeing is a powerful metaphor for the crisis of late capitalism, where exploitation, violent racialized othering, and climate denialism are the vital practices that keep the system ticking—and prevent, so far, larger and more radical popular upheavals or systemic revolutions.

## Notes

1. Any quick search on a UK news portal will easily demonstrate the overuse of that wording, for example in The Telegraph, 22 June 2024, ‘Gangs of feral youths rule our streets. Not even Waitrose is safe’ ‘https://www.telegraph.co.uk/news/2024/06/22/gangs-feral-youths-rule-our-streets-waitrose-not-safe/ (accessed 2 May 2025).

2. And even this nominal sovereignty is currently being violently unmade. We could extend the parallel to other politically divided but physically conjoined cities—East and West Berlin before 1989; Ciudad Juarez/El Paso on the US-Mexican Border; Nikosia on Cyprus—but as we argue here, the metaphor works differently in cities that are segregated in ways other than in terms of political sovereignty.

3. This is less so in Luanda, where police officers are not necessarily connotated with a provincial background. Harassment and demands for bribes are, however, equally ubiquitous.

4. Toby Kelly describes similar strategies of vehicular impression management at Israeli checkpoints in the Occupied Palestinian Territories by which Palestinian drivers seek to be “unseen” by the checkpoint guards (2006).
